# Music-related background and experience as correlates of music performance anxiety among Japanese musicians

**DOI:** 10.3389/fpsyg.2026.1763814

**Published:** 2026-05-08

**Authors:** Sakie Takagi, Akihiko Murai, Michiko Yoshie

**Affiliations:** 1 Department of Human and Engineered Environmental Studies, Graduate School of Frontier Sciences, The University of Tokyo, Kashiwa, Japan; 2 Research Institute on Human and Societal Augmentation, National Institute of Advanced Industrial Science and Technology (AIST), Kashiwa, Japan; 3 Human Informatics and Interaction Research Institute, National Institute of Advanced Industrial Science and Technology (AIST), Tsukuba, Japan; 4 Institute of Human Sciences, University of Tsukuba, Tsukuba, Japan

**Keywords:** Japanese, K-MPAI, music performance anxiety, musical experience, musician

## Abstract

Prior music performance anxiety (MPA) studies have predominantly sampled university music students. Although MPA is recognized as a global concern, evidence from Asia remains limited. To address these gaps, this study examined associations between MPA and individual attributes in a large and diverse sample of Japanese musicians aged 19–64 years across a range of musical backgrounds. We analyzed data from 390 participants who completed an online questionnaire including the Kenny Music Performance Anxiety Inventory-Revised and the Performance Anxiety Questionnaire, capturing both general dispositional aspects of MPA and performance-situation anxiety. Participants comprised 194 professional musicians and 196 amateur musicians. Multiple regression analyses were conducted to examine associations between participant attributes and MPA measures. Attendance at a music-specialized school was positively associated with MPA, whereas greater lifetime public performance experience was negatively associated with MPA. These findings suggest that the educational environment and accumulated performance experience may shape the development of MPA and should inform future research aimed at clarifying underlying mechanisms as well as the design of targeted interventions.

## 1 Introduction

Music performance anxiety (MPA) refers to a sustained form of anxious anticipation that occurs during musical performances, often developing through specific learning or conditioning (Kenny, 2009b). MPA manifests through various symptoms that can be grouped into three domains, including physiological responses such as elevated heart rate, dry mouth, or sweating, psychological difficulties including impaired concentration and memory problems, and behavioral issues such as tremors or impaired technical execution (Burin and Osório, 2017; Steptoe, 2001; Salmon, 1990; Irie et al., 2023). To explore the factors underlying MPA, previous studies have examined its associations with individual attributes.

Previous research has examined MPA across diverse contexts. Regarding age, MPA has been reported to exist from early childhood and tends to progress during adolescence (Boucher and Ryan, 2011; Osborne et al., 2005; Patston and Osborne, 2016; Dempsey and Comeau, 2019). Regarding adulthood, some studies report higher MPA among younger adults (Osborne and Franklin, 2002; Kenny et al., 2012; Butković et al., 2021), whereas others find insignificant age-related differences (Van Kemenade et al., 1995; Liston et al., 2003; Kobori et al., 2011; Dobos et al., 2018; Cohen and Bodner, 2019; Lupiáñez et al., 2021).

Gender differences in anxiety have been consistently documented in the general adult population, with women tending to report higher levels of trait anxiety than men across countries, a pattern observed repeatedly (El-Zahhar and Hocevar, 1991; Ben-Zur and Zeidner, 1988). Consistent with this broader literature, evidence on gender differences in MPA likewise tends to indicate higher MPA in women: approximately two-thirds of studies indicate that women experience higher MPA than men (Sokoli et al., 2022; Casanova et al., 2018; Coşkun-Şentürk and Çırakoğlu, 2017; Patston and Osborne, 2016; Rae and McCambridge, 2004; Iusca and Dafinoiu, 2012; Kenny et al., 2012; Liston et al., 2003; Osborne and Franklin, 2002; Osborne and Kenny, 2005). However, several studies report insignificant gender effects (Kobori et al., 2011; Fehm and Schmidt, 2006; Butković et al., 2021).

Regarding musical background, findings on years of playing experience are inconsistent, ranging from negative associations (Osborne and Franklin, 2002; Boucher and Ryan, 2011) to no relationship (Rae and McCambridge, 2004; Sadler and Miller, 2010; Kobori et al., 2011; Robson and Kenny, 2017). Music majors often report higher MPA than their counterparts (Robson and Kenny, 2017), and students generally exhibit higher anxiety than professionals (Biasutti and Concina, 2014; González et al., 2018).

Regarding performance conditions, classical musicians tend to report higher anxiety compared with jazz or popular genre musicians (Papageorgi et al., 2011), and vocalists generally exhibit lower anxiety than instrumentalists (Van Kemenade et al., 1995; Robson and Kenny, 2017; Ryan and Andrews, 2009). Studies identify solo performance as more anxiety-provoking than ensemble performance across studies (Ryan and Andrews, 2009; Papageorgi et al., 2011; Cohen and Bodner, 2019; Casanova et al., 2018). Lastly, the experience of public performance appears to influence MPA; a higher number of performances is often associated with lower MPA (Sârbescu and Dorgo, 2014; Simoens et al., 2015; Casanova et al., 2018; Coşkun-Şentürk and Çırakoğlu, 2017). From a mechanistic perspective, this pattern may be partly explained by habituation or extinction processes through repeated exposure to evaluative performance situations (Herman and Clark, 2023). Solo and public performance contexts typically involve heightened social-evaluative threat and increased personal responsibility, which may amplify anxiety responses. Repeated engagement in such contexts may, over time, attenuate threat reactivity, providing a plausible explanation for why accumulated performance experience is often associated with lower MPA. Taken together, these considerations offer a general interpretive framework for understanding why certain performance contexts are experienced as more anxiety-provoking.

Although various attributes have been examined in relation to MPA, most studies rely heavily on samples of university music majors and conservatory students, making this group disproportionately represented. While professional musicians have been explored, studies on amateur performers are relatively scarce. Geographically, existing literature on MPA remains dominated by studies conducted in Europe and Australia, resulting in limited representation of musicians from other regions. Such sampling biases may contribute to inconsistencies in reported findings and restrict the generalizability of conclusions.

In Japan, (Yoshie et al. 2011) found that about 64% of musicians experienced MPA, a prevalence broadly comparable to previous reports in other countries, for example, 57% among Turkish orchestral musicians (Topoğ lu et al., 2018) and 50% in an international survey (James, 2000). Their study also reported differences by instrument and performance context: 68.9% of pianists experienced MPA, which may reflect the relatively high frequency of solo performances, and more than 40% of wind instrument players considered practice stressful, because their sound tends to stand out even in large ensembles. More recently, (Irie et al. 2023) examined mental and physical changes surrounding performances and highlighted differences between students and professionals. Students experienced prolonged symptoms and tended to appraise them negatively, whereas professionals reported symptoms mainly close to the performance and perceived them as natural or even beneficial. Students relied on coping strategies such as self-talk and ritualized behaviors, whereas professionals used systematic, experience-based preparation. Despite these valuable contributions, research specifically focusing on Japanese musicians remains limited. Further investigation is therefore required to deepen the understanding of MPA within this population.

The present study investigates associations between MPA and individual attributes in a large sample of Japanese musicians, including both amateur and professional performers. We examine whether commonly reported correlates of MPA (e.g., gender and age) are also observed in this context and further explore performance-related factors (e.g., musical background and experience of performance).

## 2 Methods

### 2.1 Measures

#### 2.1.1 Performer Profile Questionnaire

The items were developed with reference to previous studies examining associations between individual attributes and MPA (Yoshie et al., 2011; Sokoli et al., 2022; Kenny et al., 2012). In addition, several items related to musical experience and background were added following discussion among the authors. The questionnaire included items on age and gender; music-related income, attendance at a school specializing in music, current occupation (e.g., performing musician and private-lesson instructor), attendance of private lessons, age at which participants began playing or singing, years of performance experience, number of public performances in the past five years, typical annual public-performance frequency and usual ensemble size by age range (e.g., ages 12–15 years having three performances per year in the wind band, which totaled 30 members), awards received, primary genre (e.g., classical and jazz), for instrumentalists, principal instrument and performance settings (e.g., solo and ensemble), and vocalists, performance settings, and accompanying status (e.g., accompanied or unaccompanied).

#### 2.1.2 Kenny Music Performance Anxiety Inventory Revised version

The Kenny Music Performance Anxiety Inventory (K-MPAI) is an instrument developed to evaluate anxiety symptoms and associated constructs within the domain of musical performance. Originally comprising 26 items (Kenny et al., 2004), it was subsequently revised and expanded to 40 items (K-MPAI-R) (Kenny, 2009a). The K-MPAI-R has been translated into multiple languages and is widely used in research across different countries. Accordingly, we employed the Japanese version of the K-MPAI-R (Takagi et al., 2025), a comprehensive instrument designed to assess broad, trait-like tendencies and related constructs of MPA. Further details regarding the validity of the Japanese version are provided in (Takagi et al. 2025). Participants rate each item on a seven-point Likert-type scale, with scores ranging from 0 (“strongly disagree”) to 6 (“strongly agree”). Following (Kenny 2009a), items 1, 2, 9, 17, 23, 33, 35, and 37 were reverse-scored. A total score was obtained by summing all 40 items. Additionally, factor scores were calculated as the sum of the constituent items—F1: MPA symptoms (ten items: 10, 12, 15, 16, 22, 24, 26, 30, 34, and 36); F2: psychological vulnerability (eight items: 3, 4, 6, 8, 13, 19, 20, and 31), and F3: worry/dread focused on self/other scrutiny and evaluation (six items: 18, 21, 25, 28, 38, and 39) (Takagi et al., 2025). Factors 4–7 were not used for factor-based scoring because each consisted of only two to three items. These factors reflected parental support, memory/self-efficacy, uncontrollability, and generational transmission of anxiety. This decision did not affect the K-MPAI-R total score (computed from all items). In the present sample, the K-MPAI-R showed high internal consistency (Cronbach's α = 0.93).

#### 2.1.3 Performance Anxiety Questionnaire

The Performance Anxiety Questionnaire (PAQ) (Cox and Kenardy, 1993) comprises 20 statements, with 10 reflecting cognitive aspects and 10 reflecting somatic aspects of anxiety during musical performance. It assesses how often these responses occur across three contexts, including practice, group public performance, and solo public performance. Each statement is rated on a five-point Likert-type scale from 1 (“never”) to 5 (“always”). All items were translated into Japanese using a back-translation procedure by (Kobori et al. 2011). In the present study, the PAQ was used to capture the situational frequency of anxiety responses during public performance. Participants completed the Japanese PAQ focusing on public performance, without differentiating between solo and group settings. Items 4 and 8 were reverse-scored for the PAQ in the calculation of the total score. In the present sample, the PAQ showed high internal consistency (Cronbach's α = 0.92).

### 2.2 Participants

In the present study, we reanalyzed part of the data we collected for validating the Japanese version of the K-MPAI-R (Takagi et al., 2025). Although the original sample consisted of 400 musicians, data from 10 respondents were excluded in the present study due to incomplete responses for the Performer Profile Questionnaire, leaving an analytic sample of 390 participants. Of these, 243 were men, and 147 were women. The participants were aged 19 to 64 years (M = 46.70, standard deviation [SD] = 10.52) and were native speakers of Japanese. Eligibility required current engagement in musical performance, as either an instrumental performer (*n* = 299) or vocalist (*n* = 91), and at least one public performance within the past 5 years. “Public performance” included contexts in which performances could be evaluated (e.g., examinations, competitions, and auditions) and appearances before general audiences; it did not include performances confined to family or close friends, routine practice, classes, or lessons. Participants comprised 194 professional musicians (defined as those reporting music-related income or having attended/graduated from a music-specialized school) and 196 amateur musicians.

### 2.3 Procedure

This study analyzed data obtained from an online survey. Participants were recruited via an online panel administered by a marketing research firm. Before the survey, individuals viewed an information sheet and provided electronic informed consent. Those who consented to participate completed the Performer Profile Questionnaire, K-MPAI-R, and PAQ. This study was approved by the Ethics Committee of the National Institute of Advanced Industrial Science and Technology.

### 2.4 Data analysis

All analyses were conducted on the analytic sample using complete-case analysis. Prior to the statistical analyses, residual diagnostics (residuals vs. fitted, normal Q–Q, scale–location, and residuals vs. leverage) were conducted for each outcome variable, and residual normality was assessed using the Shapiro–Wilk test. Detailed diagnostic results are provided in the Supplementary material.

Several preprocessing steps were applied to the questionnaire data. Lifetime performance counts were calculated based on participants' reports of the typical number of performances they engaged in during each age range. For example, a participant who reported three performances per year between ages 12 and 15 was assigned nine performances for that period. The reported performance counts for all age ranges were then summed to obtain each individual's lifetime total. Because both the 5-year and lifetime performance counts showed strong right-skewness, we applied a log(1+*x*) transformation to the variables. Instrument categories were coded based on participants' free-text responses: piano, guitar, strings, wind instruments, percussion, Japanese instruments, and voice. Participants initially reported their typical performance setting using six categories (solo, ensemble, wind band, orchestra, band, and other). For analysis, these were grouped into four categories according to ensemble size: solo, small ensembles of approximately 2–10 players (ensemble and band responses), large ensembles with 10 or more players (wind bands and orchestras), and others. Regarding performance awards, although the survey asked about regional, prefectural, national, and international prizes separately, participants were classified as “Yes” if they had received at least one award in any category.

To screen candidate predictors and determine the order of variable entry for multivariable model selection, we first conducted univariate analyses for each predictor–outcome pair. Regarding continuous predictors (age, start age, years of experience, and performance counts), simple linear regression models were fitted and unstandardized slopes, 95% confidence intervals (CIs), and *p*-values were reported. Regarding binary predictors, Welch's *t*-tests were used. Regarding multi-level categorical predictors, omnibus group differences were evaluated using one-way analysis of variance.

Before constructing multivariable models, we assessed multicollinearity among candidate predictors. Variance inflation factors (VIFs) were all within acceptable limits, indicating no problematic collinearity (all VIF < 4.5); detailed values are presented in the Supplementary material.

We further constructed multiple regression models for each outcome (K-MPAI-R total and subfactors and PAQ). Candidate predictors consisted of variables that were retained after the univariate screening stage for each outcome (*p* < 0.05). Model selection followed a forward-selection procedure maximizing adjusted *R*^2^. Predictors were added in ascending order of univariate *p*-values and retained only if they improved adjusted *R*^2^. Robust standard errors (HC3) were used to account for heteroskedasticity, and *p*-values were adjusted within each outcome using the Benjamini–Hochberg procedure. Final models report unstandardized and standardized coefficients, 95% CIs, and adjusted *p*-values. Model stability was additionally evaluated using bootstrap resampling; full details and results are provided in the Supplementary material.

All analyses were conducted in R (R Core Team, 2025) with the following packages: sandwich (Zeileis et al., 2020; Zeileis, 2004), lmtest (Zeileis and Hothorn, 2002), car (Fox and Weisberg, 2019), broom (v1.0.9; Robinson et al., 2025), and emmeans (v1.11.2; Lenth, 2025).

## 3 Results

### 3.1 Descriptive analysis

Table 1 summarizes the descriptive statistics (mean, SD, and range) for all outcome scores (K-MPAI-R total and factors, and PAQ). Table 2 reports the descriptive statistics for continuous attributes (age, start age, years of experience, and performance counts). Table 3 presents the distribution of categorical attributes, including gender, music-related income, education, occupation, genre, instrument category, and performance setting.

**Table 1:** Descriptive statistics of outcome scores.

Variable	Mean	SD	Min	Max
K–MPAI–R total	109.35	34.47	12	207
F1: MPA symptoms	24.74	12.11	0	60
F2: Psychological vulnerability	20.90	10.16	0	48
F3: Worry/Dread (scrutiny/evaluation)	16.62	8.19	0	36
PAQ total	54.42	14.50	21	96

**Table 2:** Descriptive statistics of continuous attributes.

Variable	Mean	SD	Min	Max
Age	46.70	10.52	19	64
Start age	16.58	10.86	1	60
Years of performance experience	26.43	14.39	1	60
Performance count (past 5 years)	25.05	82.05	1	1,000
Performance count (lifetime)	164.10	842.51	1	13,240

**Table 3:** Distribution of categorical participant attributes.

Attribute	Level	*n*	%
Gender	Men	243	62.3%
	Women	147	37.7%
Music-related income	Yes	144	36.9%
	No	246	63.1%
Attendance at a music-specialized school	Yes	154	39.5%
	No	236	60.5%
Occupation	Performing musician	59	15.1%
	Music faculty (college/vocational)	16	4.1%
	Private lesson instructor	68	17.4%
	Music teacher (elementary–high school)	46	11.8%
	Music student (college/vocational)	8	2.1%
	Others	246	63.1%
	Attendance at private lessons	Yes	110	28.2%
No		280	71.8%
Awards	Yes	232	59.5%
	No	158	40.5%
Primary genre	Classical	160	41.0%
	Jazz	28	7.2%
	Rock	59	15.1%
	Pops	73	18.7%
	Others	70	17.9%
Instrument category	Piano	113	29.0%
	Guitar	71	18.2%
	String	12	3.1%
	Wind Instrument	64	16.4%
	Percussion	26	6.7%
	Japanese Instrument	13	3.3%
	Voice	91	23.3%
Performance setting	Solo	189	48.5%
	2-10 people	120	30.8%
	10- people	74	19.0%
	Others	7	1.8%

### 3.2 Univariate associations

#### 3.2.1 K-MPAI-R

We first examined univariate associations between participant attributes and each outcome. Regarding the total score, significant predictors included age, years of performance experience, performance count (lifetime), gender, music-related income, attendance at a music-specialized school, and awards. Regarding Factor 1 (MPA symptoms), significant predictors included age, years of performance experience, performance count (lifetime), gender, music-related income, attendance at a music-specialized school, attendance at private lessons, awards, primary genre, and performance setting. Regarding Factor 2 (psychological vulnerability), significant predictors included age, years of performance experience, performance count (lifetime), attendance at a music-specialized school, and awards. Regarding Factor 3 (worry/dread), significant predictors included age, gender, music-related income, attendance at a music-specialized school, attendance at private lessons, primary genre, instrument category, and performance setting. Complete statistics are presented in the Supplementary material.

#### 3.2.2 PAQ

Univariate associations with the PAQ total score are summarized in the Supplementary material. Significant predictors included age, years of performance experience, performance count (lifetime), gender, music-related income, attendance at a music-specialized school, attendance at private lessons, and performance setting.

### 3.3 Multiple regression

#### 3.3.1 K-MPAI-R

A forward stepwise multiple regression analysis was conducted to identify the predictors of K-MPAI-R total scores. The screened predictors were attendance at a school specializing in music, age, lifetime public performance count, gender, music-related income, years of performance experience, and awards received. The model explained *R*^2^ = 0.098 of the variance ($R_{\text{adj}}^{2}=0.088$; *n* = 390). After controlling for multiple comparisons using the Benjamini–Hochberg procedure, significant predictors were attendance at a music-specialized school (*B* = 11.97, *q*_BH_ < 0.01) and lifetime performance count (*B* = −3.75, *q*_BH_ < 0.01). Musicians who attended music-specialized schools reported higher overall anxiety scores. However, those with more performance experience reported lower scores. Other predictors, including age, gender, and music-related income, did not remain significant after correction. Table 4 presents the detailed results.

**Table 4:** Multiple regression for K-MPAI-R total.

Predictor	B	b	SE	95% CI	*p*	qBH
Attended music-specialized school: Yes (vs. No)	11.97	0.17	4.00	[4.10, 19.84]	**< 0.01**	**< 0.01**
Age	–0.35	–0.11	0.18	[–0.70, 0.00]	0.052	0.078
Performance count (all-time, log)	–3.75	–0.18	1.18	[–6.06, -1.44]	**< 0.01**	**< 0.01**
Gender: Women (vs. Men)	4.16	0.06	3.72	[–3.14, 11.47]	0.263	0.263
Music-related income: Yes (vs. No)	5.56	0.08	4.32	[–2.93, 14.05]	0.198	0.238

B, unstandardized regression coefficient; b, standardized regression coefficient; SE, standard error; 95% CI, 95% confidence interval; p, p value; qBH, Benjamini-Hochberg adjusted p value. Bold values indicate statistically significant p-values and BH-adjusted p-values.

Regarding F1 (MPA symptoms), the screened predictors were age, attendance at a school specializing in music, awards received, lifetime public performance count, gender, years of performance experience, music-related income, usual ensemble size, primary genre, and attendance at private lessons. The model explained *R*^2^ = 0.158 of the variance ($R_{\text{adj}}^{2}=0.127$; *n* = 390). After controlling for multiple comparisons using the Benjamini–Hochberg procedure, significant predictors were age (*B* = −0.19, *q*_BH_ < 0.05) and lifetime performance count (*B* = −1.40, *q*_BH_ < 0.05), indicating that younger musicians reported higher symptom scores, whereas more performance experience predicted lower symptoms. Regarding F2 (psychological vulnerability), the screened predictors were attendance at a school specializing in music, age, lifetime public performance count, years of performance experience, and awards received. The model explained *R*^2^ = 0.081 of the variance ($R_{\text{adj}}^{2}=0.071$; *n* = 390), and significant predictors were attendance at a music-specialized school (*B* = 3.81, *q*_BH_ < 0.001) and lifetime performance count (*B* = −0.89, *q*_BH_ < 0.05), suggesting that musicians who attended a music-specialized school reported higher vulnerability while those with more performance experience reported lower vulnerability. Regarding F3 (worry/dread), the screened predictors for Factor 3 were attendance at a school specializing in music, music-related income, attendance at private lessons, gender, usual ensemble size, principal instrument category, age, and primary genre. The model explained *R*^2^ = 0.112 of the variance ($R_{\text{adj}}^{2}=0.078$; *n* = 390), but no predictors remained significant after multiple comparison correction. Tables 5–7 present the detailed results.

**Table 5:** Multiple regression for F1 (MPA symptoms).

Predictor	B	b	SE	95% CI	*p*	qBH
Age	–0.19	–0.16	0.06	[–0.30, –0.07]	**< 0.01**	**< 0.05**
Attended music-specialized school: Yes (vs. No)	3.01	0.12	1.49	[0.07, 5.95]	**< 0.05**	0.168
Awards: Yes (vs. No)	2.00	0.08	1.27	[–0.50, 4.51]	0.116	0.283
Performance count (all-time, log)	–1.40	–0.19	0.45	[–2.29, –0.51]	**< 0.01**	**< 0.05**
Gender: Women (vs. Men)	0.95	0.04	1.39	[–1.79, 3.69]	0.496	0.677
Music-related income: Yes (vs. No)	2.63	0.11	1.58	[–0.47, 5.73]	0.096	0.283
Performance setting: 2–10 (vs. Solo)	–1.76	–0.07	1.55	[–4.80, 1.28]	0.256	0.428
Performance setting: 10+ (vs. Solo)	0.99	0.03	1.67	[–2.29, 4.27]	0.555	0.694
Performance setting: Others (vs. Solo)	6.14	0.07	6.73	[–7.10, 19.38]	0.363	0.544
Primary genre: Jazz (vs. Classical)	0.14	0.00	2.72	[–5.21, 5.49]	0.959	0.959
Primary genre: Rock (vs. Classical)	0.78	0.02	2.14	[–3.43, 4.98]	0.717	0.827
Primary genre: Pops (vs. Classical)	0.13	0.00	1.65	[–3.10, 3.36]	0.937	0.959
Primary genre: Others (vs. Classical)	-2.71	-0.09	1.80	[–6.25, 0.82]	0.132	0.283
Private lessons: Yes (vs. No)	1.60	0.06	1.41	[–1.17, 4.38]	0.257	0.428

B, unstandardized regression coefficient; b, standardized regression coefficient; SE, standard error; 95% CI, 95% confidence interval; p, p value; qBH, Benjamini-Hochberg adjusted p value. Bold values indicate statistically significant p-values and BH-adjusted p-values.

**Table 6:** Multiple regression for F2 (Psychological vulnerability).

Predictor	B	b	SE	95% CI	*p*	qBH
Attended music-specialized school: Yes (vs. No)	3.81	0.18	1.06	[1.73, 5.90]	**< 0.001**	**< 0.001**
Age	–0.09	–0.10	0.05	[–0.20, 0.01]	0.065	0.081
Performance count (all-time, log)	–0.89	–0.14	0.34	[–1.55, –0.23]	**< 0.01**	**< 0.05**
Awards: Yes (vs. No)	1.45	0.07	1.06	[–0.64, 3.54]	0.174	0.174

B, unstandardized regression coefficient; b, standardized regression coefficient; SE, standard error; 95% CI, 95% confidence interval; p, p value; qBH, Benjamini-Hochberg adjusted p value. Bold values indicate statistically significant p-values and BH-adjusted p-values.

**Table 7:** Multiple regression for F3 (Worry/Dread).

Predictor	B	b	SE	95% CI	*p*	qBH
Attended music-specialized school: Yes (vs. No)	1.59	0.10	1.07	[–0.51, 3.69]	0.138	0.339
Music-related income: Yes (vs. No)	1.86	0.11	1.09	[–0.27, 4.00]	0.087	0.262
Private lessons: Yes (vs. No)	2.00	0.11	0.97	[0.10, 3.91]	**< 0.05**	0.194
Gender: Women (vs. Men)	0.03	0.00	0.96	[–1.85, 1.91]	0.977	0.977
Performance setting: 2–10 (vs. Solo)	–1.42	–0.08	1.00	[–3.39, 0.55]	0.158	0.339
Performance setting: 10+ (vs. Solo)	0.98	0.05	1.37	[–1.72, 3.67]	0.477	0.565
Performance setting: Others (vs. Solo)	7.26	0.12	3.64	[0.11, 14.41]	**< 0.05**	0.194
Instrument category: Guitar (vs. Piano)	–1.36	-0.06	1.31	[–3.93, 1.21]	0.300	0.450
Instrument category: String (vs. Piano)	–1.92	-0.04	2.04	[–5.95, 2.10]	0.347	0.473
Instrument category: Wind (vs. Piano)	–0.21	-0.01	1.52	[–3.21, 2.78]	0.890	0.954
Instrument category: Percussion (vs. Piano)	2.37	0.07	1.98	[–1.52, 6.27]	0.232	0.386
Instrument category: Japanese (vs. Piano)	–5.60	–0.12	2.87	[–11.24, 0.04]	0.052	0.194
Instrument category: Voice (vs. Piano)	–0.78	–0.04	1.14	[-3.02, 1.45]	0.490	0.565
Age	–0.06	–0.07	0.04	[–0.14, 0.03]	0.187	0.350

B, unstandardized regression coefficient; b, standardized regression coefficient; SE, standard error; 95% CI, 95% confidence interval; p, p value; qBH, Benjamini-Hochberg adjusted p value. Bold values indicate statistically significant p-values and BH-adjusted p-values.

#### 3.3.2 PAQ

The screened predictors for the PAQ total score were attendance at a school specializing in music, lifetime public performance count, age, attendance at private lessons, gender, years of performance experience, usual ensemble size, and music-related income. The model explained *R*^2^ = 0.136 of the variance ($R_{\text{adj}}^{2}=0.125$; *n* = 390). After controlling for multiple comparisons using the Benjamini–Hochberg procedure, significant predictors were attendance at a music-specialized school (*B* = 6.68, *q*_BH_ < 0.001) and log-transformed lifetime performance count (*B* = −2.11, *q*_BH_ < 0.001). Musicians who attended a music-specialized school reported higher performance anxiety questionnaire scores, whereas those with more performance experience reported lower scores. Other predictors, including attendance at private lessons, age, and gender, did not remain significant after correction. Table 8 presents the complete statistics.

**Table 8:** Multiple regression for PAQ.

Predictor	B	b	SE	95% CI	*p*	qBH
Attended music-specialized school: Yes (vs. No)	6.68	0.23	1.41	[3.90, 9.46]	**< 0.001**	**< 0.001**
Performance count (all-time, log)	–2.11	–0.24	0.46	[–3.01, –1.21]	**< 0.001**	**< 0.001**
Age	–0.09	–0.06	0.07	[–0.23, 0.05]	0.215	0.215
Private lessons: Yes (vs. No)	3.26	0.10	1.58	[0.16, 6.37]	**< 0.05**	0.059
Gender: Women (vs. Men)	1.91	0.06	1.51	[–1.05, 4.87]	0.205	0.215

B, unstandardized regression coefficient; b, standardized regression coefficient; SE, standard error; 95% CI, 95% confidence interval; p, p value; qBH, Benjamini-Hochberg adjusted p value. Bold values indicate statistically significant p-values and BH-adjusted p-values.

## 4 Discussion

This study examines individual differences in MPA among Japanese musicians using a sample of 390 participants that included both amateur and professional performers. Participants completed MPA measures, including the K-MPAI-R and PAQ, as well as the Performer Profile Questionnaire assessing demographic and musical background attributes. Forward stepwise multiple regression analyses were conducted with MPA variables and profile-based predictors as the dependent and independent variables, respectively. Across all models, two factors consistently emerged as significant after controlling for multiple comparisons: specialized music education and lifetime performance experience.

For reference, the mean K-MPAI-R total score in the present Japanese sample (M = 109.35, SD = 34.47) was comparable to those reported in European conservatoire student samples, such as those from Italy (M = 111.66, SD = 40.87) (Antonini Philippe et al., 2023) and Lithuania (M = 95.27, SD = 34.89) (Paliaukiene et al., 2018). However, these values should be interpreted cautiously, as prior studies predominantly focused on university-level music students, whereas the present sample included both amateur and professional musicians.

One of the most notable findings was the association between specialized music education and elevated MPA. After controlling for multiple comparisons, attendance at a music-specialized school remained a significant predictor for the K-MPAI-R total score, Factor 2 of K-MPAI-R, and PAQ, indicating that musicians with this educational background reported higher anxiety compared with their counterparts. Previous research indicated that students enrolled in specialized music programs are repeatedly exposed to high-pressure assessments, such as juried examinations, solo recitals, competitions, and auditions, where their performances are closely scrutinized and evaluated (Okan and Usta, 2021; Robson and Kenny, 2017). Similarly, in the context of Japan, specialized music education entails frequent exposure to evaluative environments such as individual lessons, performance exams, and internal auditions, mirroring Western conservatory systems (Kishi, 2022). This training pathway can also be highly selective; for example, at the Tokyo University of the Arts (a national university in Japan), the applicant-to-seat ratio for the Faculty of Music is commonly reported to be 3–5 applicants per place. Students—particularly those in piano and string disciplines—often enter competitions at an early age, where the results can influence their educational and mentorship opportunities. They must also make career-defining choices at multiple stages of schooling. Despite this early specialization, only a few students succeed as professional musicians, and even those often face economic instability, relying on teaching alongside performance to sustain their careers (Ogawa, 2021). Together, these features suggest that sustained exposure to selective and strongly evaluative training during adolescence and early adulthood may be linked to higher MPA later in life. Nevertheless, specialized music education provides a crucial context in which effective coping strategies for MPA can be systematically integrated throughout the training pathway, from early instruction to professional preparation. By embedding psychoeducation on the physical and psychological demands of performance, including an understanding of the body's stress responses, as well as empirically supported practice and performance strategies to manage these demands within curricula, educational institutions may better equip musicians to perform in ways that are both healthy and sustainable (Herman and Clark, 2023; Matei et al., 2018).

Another key finding was the negative association between lifetime performance count and MPA, which remained significant after controlling for multiple comparisons for the K-MPAI-R total score, F1, F2, and PAQ total score. This pattern is consistent with previous studies showing that more frequent performance is related to reduced MPA (Sârbescu and Dorgo, 2014; Simoens et al., 2015; Coşkun-Şentürk and Çırakoğlu, 2017), with the suggestion that limited performance experience may restrict opportunities to develop effective strategies to manage performance-related anxiety (Casanova et al., 2018).

Age showed a modest association with MPA, particularly for Factor 1 of the K-MPAI-R. After correction, older participants reported lower scores on this factor, suggesting greater resilience against physiological and cognitive symptoms of anxiety. This pattern aligns with evidence that younger adults tend to experience higher MPA (Osborne and Franklin, 2002; Kenny et al., 2012; Butković et al., 2021). Although the reasons for this pattern remain unclear, several possibilities can be considered. Trait anxiety tends to be higher among younger adults (Spielberger et al., 1970). Similarly, data from Japan indicate that both state and trait anxiety decline from early adulthood until old age (Nakazato and Shimonaka, 1989). Additionally, age-related changes in neuroendocrine function and emotion regulation may contribute to reduced physiological reactivity to performance stress among older adults, for example, through a decline in stress-related cortisol responsiveness (Mikneviciute et al., 2023) and shifts in amygdala–ventral prefrontal cortex structural connectivity (Clewett et al., 2014). These explanations remain tentative, and further research is needed to clarify whether age-related differences in MPA reflect developmental factors, cohort effects, or other unmeasured influences.

In the univariate analyses, women reported significantly higher scores than men on the K-MPAI-R total score, Factors 1 and 3, and PAQ. This pattern is consistent with prior research showing higher anxiety-related scores among women (Sokoli et al., 2022; Casanova et al., 2018; Coşkun-Şentürk and Çırakoğlu, 2017; Patston and Osborne, 2016; Rae and McCambridge, 2004; Iusca and Dafinoiu, 2012; Kenny et al., 2012; Liston et al., 2003; Osborne and Franklin, 2002; Osborne and Kenny, 2005), and the present findings suggest that a similar tendency is observable in Japanese musicians. However, gender did not remain a significant predictor in the multivariable models, indicating that the observed gender differences in the univariate analyses may partly be attributable to other correlated individual attributes rather than an independent effect.

Other variables, including music-related income, attendance at private lessons, performance setting, primary genre, and instrument category, did not remain significant after controlling for multiple comparisons in the regression models. Although some of these factors were associated in unadjusted analyses, their effects were not robust when considered alongside other predictors. This suggests that their influence on MPA may be limited or context dependent, and future research should explore potential interactions or mediating mechanisms rather than direct effects.

This study has several limitations. First, it remains unclear whether the findings reflect cultural characteristics specific to Japan or whether similar patterns would be observed in Western contexts. Our survey design did not include a cross-cultural comparison; therefore, we could not determine the extent to which the associations identified here are culture-specific. Future studies should conduct comparative investigations focusing on the key variables identified in this study. Second, the observed associations should be interpreted as correlational and do not permit causal inference or clear conclusions regarding directionality. Longitudinal and/or experimental studies, ideally incorporating objective indicators, are needed to clarify temporal ordering and underlying mechanisms. Third, because responses were collected via an online survey, musicians with limited digital access may have been less likely to participate, potentially introducing selection bias. Finally, our sample included a large proportion of classical musicians in terms of musical genre, a relatively small proportion of students in terms of occupation, and string players were underrepresented among instrumentalists. These sampling biases should be considered when interpreting the results and addressed in future research.

In summary, this study conducted a comprehensive survey of Japanese musicians and examined the associations between MPA and individual attributes. The results suggest that attendance at a music-specialized school and lifetime performance count are key correlates of MPA in this population. Building on these findings, future research should clarify the mechanisms by which these background factors contribute to the development and maintenance of MPA, and develop effective coping strategies and interventions for musicians.

## Data Availability

The deidentified questionnaire data supporting the conclusions of this article will be made available by the corresponding authors upon request. Requests to access these datasets should be directed to Sakie Takagi, sakie.takagi@aist.go.jp; Michiko Yoshie, m.yoshie@aist.go.jp.

## References

[B1] Antonini Philippe R. Cruder C. Biasutti M. Crettaz Von Roten F. (2023). The Kenny Music Performance Anxiety Inventory-Revised (K-MPAI-R): validation of the Italian version. Psychol. Music 51, 565–578. doi: 10.1177/03057356221101430

[B2] Ben-Zur H. Zeidner M. (1988). Sex differences in anxiety, curiosity, and anger: a cross-cultural study. Sex Roles 19, 335–347. doi: 10.1007/BF00289841

[B3] Biasutti M. Concina E. (2014). The role of coping strategy and experience in predicting music performance anxiety. Musicae Sci. 18, 189–202. doi: 10.1177/1029864914523282

[B4] Boucher H. Ryan C. A. (2011). Performance stress and the very young musician. J. Res. Music Educ. 58, 329–345. doi: 10.1177/0022429410386965

[B5] Burin A. B. Osório F. L. (2017). Music performance anxiety: a critical review of etiological aspects, perceived causes, coping strategies and treatment. Arch. Clin. Psychiatr. 44, 127–133. doi: 10.1590/0101-60830000000136

[B6] Butković A. Vukojević N. Carević S. (2021). Music performance anxiety and perfectionism in Croatian musicians. Psychol. Music 50, 100–110. doi: 10.1177/0305735620978692

[B7] Casanova O. Zarza F. J. Orejudo S. (2018). Differences in performance anxiety levels among advanced conservatory students in Spain, according to type of instrument and academic year of enrolment. Music. Educ. Res. 20, 377–389. doi: 10.1080/14613808.2018.1433145

[B8] Clewett D. Bachman S. Mather M. (2014). Age-related reduced prefrontal-amygdala structural connectivity is associated with lower trait anxiety. Neuropsychology 28, 631–642. doi: 10.1037/neu0000060 24635708 PMC4087066

[B9] Cohen S. Bodner E. (2019). Flow and music performance anxiety: the influence of contextual and background variables. Music Sci. 25, 25–44. doi: 10.1177/1029864919838600

[B10] Coşkun-Şentürk G. Çırakoğlu O. C. (2017). How guilt/shame proneness and coping styles are related to music performance anxiety and stress symptoms by gender. Psychol. Music 46, 682–698. doi: 10.1177/0305735617721338

[B11] Cox W. J. Kenardy J. (1993). Performance anxiety, social phobia, and setting effects in instrumental music students. J. Anxiety Disord. 7, 49–60. doi: 10.1016/0887-6185(93)90020-L

[B12] Dempsey E. Comeau G. (2019). Music performance anxiety and self-efficacy in young musicians: effects of gender and age. MPR 9, 60–79.

[B13] Dobos B. Piko B. F. Kenny D. T. (2018). Music performance anxiety and its relationship with social phobia and dimensions of perfectionism. Res. Stud. Music Educ. 41, 310–326. doi: 10.1177/1321103X18804295

[B14] El-Zahhar N. E. Hocevar D. (1991). Cultural and sexual differences in test anxiety, trait anxiety and arousability: Egypt, brazil, and the united states. J. Cross Cult. Psychol. 22, 238–249. doi: 10.1177/0022022191222005

[B15] Fehm L. Schmidt K. (2006). Performance anxiety in gifted adolescent musicians. J. Anxiety Disord. 20, 98–109. doi: 10.1016/j.janxdis.2004.11.011 16325117

[B16] Fox J. Weisberg S. (2019). An R Companion to Applied Regression, 3rd Edn. Thousand Oaks, CA: Sage.

[B17] González A. Blanco-Pi neiro P. Díaz-Pereira M. P. (2018). Music performance anxiety: exploring structural relations with self-efficacy, boost, and self-rated performance. Psychol. Music 46, 831–847. doi: 10.1177/0305735617727822

[B18] Herman R. Clark T. (2023). It's not a virus! Reconceptualizing and de-pathologizing music performance anxiety. Front. Psychol. 14:1194873. doi: 10.3389/fpsyg.2023.1194873 38022988 PMC10667921

[B19] Irie N. Morijiri Y. Yoshie M. (2023). Symptoms of and coping strategies for music performance anxiety through different time periods. Front. Psychol. 14:1138922. doi: 10.3389/fpsyg.2023.1138922 37325759 PMC10264607

[B20] Iusca D. Dafinoiu I. (2012). Performance anxiety and musical level of undergraduate students in exam situations: the role of gender and musical instrument. Procedia Soc. Behav. Sci. 33, 448–452. doi: 10.1016/j.sbspro.2012.01.161

[B21] James I. M. (2000). “Survey of orchestras,” in Medical Problems of the Instrumentalist Musician, eds. R. Tubiana and P. C. Amadio (London: Martin Dunitz), 195–201. doi: 10.1201/b14694-10

[B22] Kenny D. Driscoll T. Ackermann B. (2012). Psychological well-being in professional orchestral musicians in Australia: a descriptive population study. Psychol. Music 42, 210–232. doi: 10.1177/0305735612463950

[B23] Kenny D. T. (2009a). “The factor structure of the Revised Kenny Music Performance Anxiety Inventory,” in International Symposium on Performance Science (Utrecht: Association Européenne des Conservatoires), 37–41.

[B24] Kenny D. T. (2009b). “Negative emotions in music making: performance anxiety,” in Handbook of Music and Emotion: Theory, Research, Applications, eds. P. Juslin and J. Sloboda (Oxford: Oxford University Press). doi: 10.1093/acprof:oso/9780199230143.003.0016

[B25] Kenny D. T. Davis P. Oates J. (2004). Music performance anxiety and occupational stress amongst opera chorus artists and their relationship with state and trait anxiety and perfectionism. J. Anxiety Disord. 18, 757–777. doi: 10.1016/j.janxdis.2003.09.004 15474851

[B26] Kishi A. (2022). The meaning of “university” for students in music colleges: based on interviews with current and former students. Sonoda Womens Univ. J. Acad. Res. 56, 1–19.

[B27] Kobori O. Yoshie M. Kudo K. Ohtsuki T. (2011). Traits and cognitions of perfectionism and their relation with coping style, effort, achievement, and performance anxiety in Japanese musicians. J. Anxiety Disord. 25, 674–679. doi: 10.1016/j.janxdis.2011.03.001 21477982

[B28] Lenth R. V. (2025). emmeans: Estimated Marginal Means, aka Least-Squares Means. R package version 1.11.12.

[B29] Liston M. Frost A. A. M. Mohr P. B. (2003). The prediction of musical performance anxiety. MPPA 18, 120–125. doi: 10.21091/mppa.2003.3021

[B30] Lupiáñez M. Ortiz F. D. P. Vila J. Muñoz M. A. (2021). Predictors of music performance anxiety in conservatory students. Psychol. Music 50, 1005–1022. doi: 10.1177/03057356211032290

[B31] Matei R. Broad S. Goldbart J. Ginsborg J. (2018). Health education for musicians. Front. Psychol. 9:1137. doi: 10.3389/fpsyg.2018.01137 30061850 PMC6055059

[B32] Mikneviciute G. Pulopulos M. M. Allaert J. Armellini A. Rimmele U. Kliegel M. . (2023). Adult age differences in the psychophysiological response to acute stress. Psychoneuroendocrinology 153:106111. doi: 10.1016/j.psyneuen.2023.106111 37075654

[B33] Nakazato K. Shimonaka Y. (1989). A cross-sectional study of development of anxiety in adult lifespan. Jpn. J. Educ. Psychol. 37, 172–178. doi: 10.5926/jjep1953.37.2_172

[B34] Ogawa M. (2021). Music education for “musically talented children” in Japan: a career path toward professionals and three music education organizations. IJSSAI 1, 7–17. doi: 10.35745/ijssai2021v01.01.0003

[B35] Okan H. Usta B. (2021). Conservatory students' music performance anxiety and educational expectations: a qualitative study. Asian J. Educ. Train. 7, 250–259. doi: 10.20448/journal.522.2021.74.250.259

[B36] Osborne M. S. Franklin J. (2002). Cognitive processes in music performance anxiety. Aust. J. Pyschol. 54, 86–93. doi: 10.1080/00049530210001706543

[B37] Osborne M. S. Kenny D. T. (2005). Development and validation of a music performance anxiety inventory for gifted adolescent musicians. J. Anxiety Disord. 19, 725–751. doi: 10.1016/j.janxdis.2004.09.002 16076421

[B38] Osborne M. S. Kenny D. T. Holsomback R. (2005). Assessment of music performance anxiety in late childhood: a validation study of the Music Performance Anxiety Inventory for Adolescents (MPAI-A). Int. J. Stress Manag. 12, 312–330. doi: 10.1037/1072-5245.12.4.312

[B39] Paliaukiene V. Kazlauskas E. Eimontas J. Skeryte-Kazlauskiene M. (2018). Music performance anxiety among students of the academy in Lithuania. Music Educ. Res. 20, 390–397. doi: 10.1080/14613808.2018.1445208

[B40] Papageorgi I. Creech A. Welch G. (2011). Perceived performance anxiety in advanced musicians specializing in different musical genres. Psychol. Music 41, 18–41. doi: 10.1177/0305735611408995

[B41] Patston T. Osborne M. S. (2016). The developmental features of music performance anxiety and perfectionism in school age music students. Perform. Enhanc. Health 4, 42–49. doi: 10.1016/j.peh.2015.09.003

[B42] R Core Team (2025). R: A Language and Environment for Statistical Computing. Vienna: R Foundation for Statistical Computing.

[B43] Rae G. McCambridge K. (2004). Correlates of performance anxiety in practical music exams. Psychol. Music 32, 432–439. doi: 10.1177/0305735604046100

[B44] Robinson D. Hayes A. Couch S. (2025). broom: Convert Statistical Objects into Tidy Tibbles. R package version 1.0.9.

[B45] Robson K. E. Kenny D. T. (2017). Music performance anxiety in ensemble rehearsals and concerts: a comparison of music and non-music major undergraduate musicians. Psychol. Music 45, 868–885. doi: 10.1177/0305735617693472

[B46] Ryan C. Andrews N. (2009). An investigation into the choral singer's experience of music performance anxiety. J. Res. Music Educ. 57, 108–126. doi: 10.1177/0022429409336132

[B47] Sadler M. E. Miller C. J. (2010). Performance anxiety: a longitudinal study of the roles of personality and experience in musicians. Soc. Psychol. Pers. Sci. 1, 280–287. doi: 10.1177/1948550610370492

[B48] Salmon P. G. (1990). A psychological perspective on musical performance anxiety: a review of the literature. Med. Probl. Perform. Art. 5, 2–11.

[B49] Sârbescu P. Dorgo M. (2014). Frightened by the stage or by the public? Exploring the multidimensionality of music performance anxiety. Psychol. Music 42, 568–579. doi: 10.1177/0305735613483669

[B50] Simoens V. L. Puttonen S. Tervaniemi M. (2015). Are music performance anxiety and performance boost perceived as extremes of the same continuum? Psychol. Music 43, 171–187. doi: 10.1177/0305735613499200

[B51] Sokoli E. Hildebrandt H. Gomez P. (2022). Classical music students' pre-performance anxiety, catastrophizing, and bodily complaints vary by age, gender, and instrument and predict self-rated performance quality. Front. Psychol. 13:905680. doi: 10.3389/fpsyg.2022.905680 35814093 PMC9263585

[B52] Spielberger C. D. Gorsuch R. L. Lushene R. E. (1970). STAI Manual for the State-Trait Anxiety Inventory (“*Self-Evaluation Questionnaire*”). Saint Paul, MN: Consulting Psychologists Press.

[B53] Steptoe A. (2001). “Negative emotions in music making: the problem of performance anxiety,” in Music and Emotion: Theory and Research (Oxford: Oxford University Press), 291–307. doi: 10.1093/oso/9780192631886.003.0013

[B54] Takagi S. Yoshie M. Murai A. (2025). Validation of the Japanese version of the Kenny Music Performance Anxiety Inventory-Revised. Front. Psychol. 16:1543958. doi: 10.3389/fpsyg.2025.1543958 40606897 PMC12213870

[B55] Topoğlu O. Karagülle D. Keskin T. Abacigil F. Okyay P. (2018). General health status, music performance anxiety, and coping methods of musicians working in Turkish state symphony orchestras: a cross-sectional study. MPPA 33, 118–123. doi: 10.21091/mppa.2018.2019 29868686

[B56] Van Kemenade J. F. L. M. Van Son M. J. M. Van Heesch N. C. A. (1995). Performance anxiety among professional musicians in symphonic orchestras: a self-report study. Psychol. Rep. 77, 555–562. doi: 10.2466/pr0.1995.77.2.555 8559881

[B57] Yoshie M. Kanazawa E. Kudo K. Ohtsuki T. Nakazawa K. (2011). “Music performance anxiety and occupational stress among classical musicians,” in Handbook of Stress in the Occupations, eds. J. Langan-Fox and C. Cooper (Cheltenham: Edward Elgar Publishing), 409–425. doi: 10.4337/9780857931153.00051

[B58] Zeileis A. (2004). Econometric computing with HC and HAC covariance matrix estimators. J. Stat. Softw. 11, 1–17. doi: 10.18637/jss.v011.i10

[B59] Zeileis A. Hothorn T. (2002). Diagnostic checking in regression relationships. R News 2, 7–10.

[B60] Zeileis A. Köll S. Graham N. (2020). Various versatile variances: an object-oriented implementation of clustered covariances in R. J. Stat. Softw. 95, 1–36. doi: 10.18637/jss.v095.i01

