# Supplementary material for: Music-related background and experience as correlates of music performance anxiety among Japanese musicians
Source: Front Psychol. 2026 May 8;17:1763814. doi: 10.3389/fpsyg.2026.1763814 (PMC13194396; doi:10.3389/fpsyg.2026.1763814)
Supplement: Supplementary file 1 [file Data_Sheet_1.docx]

Supplementary Material

Music-related background and experience as correlates of music performance anxiety among Japanese musicians

Sakie Takagi^*^, Akihiko Murai, and Michiko Yoshie^*^.

* Correspondence Sakie Takagi [sakie.takagi@aist.go.jp](mailto:sakie.takagi@aist.go.jp) and Michiko Yoshie [m.yoshie@aist.go.jp](mailto:m.yoshie@aist.go.jp)

# Supplementary Figures


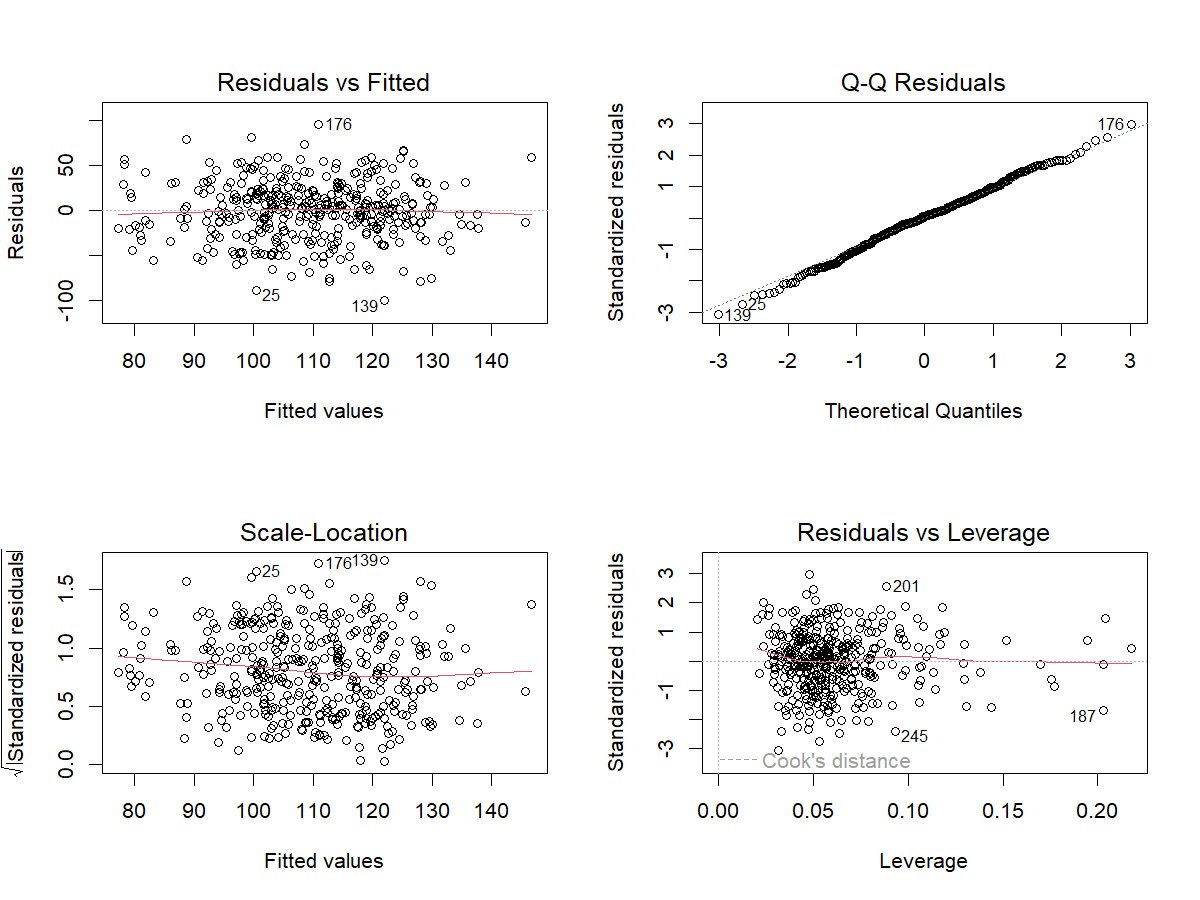


Supplementary Figure 1. Residual diagnostic plots for the Kenny Music Performance Anxiety Inventory-Revised total score

Each panel shows standard residual diagnostic plots (residuals vs. fitted values, normal Q–Q plot, scale–location plot, and residuals vs. leverage) for the fitted linear model. Normality of residuals was additionally evaluated using the Shapiro–Wilk test. The Shapiro–Wilk test did not indicate a significant deviation from normality (W = 0.53, p = .527).


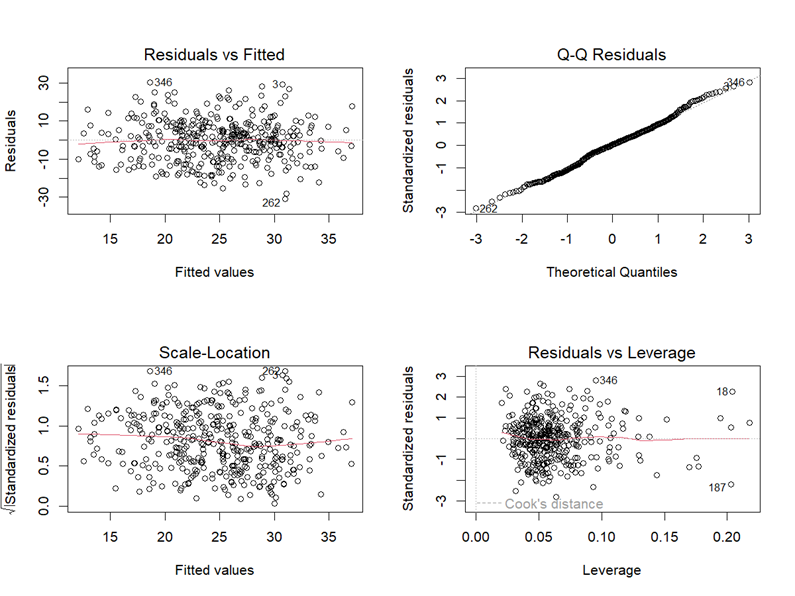


Supplementary Figure 2. Residual diagnostic plots for the Kenny Music Performance Anxiety Inventory-Revised Factor 1

Each panel shows standard residual diagnostic plots (residuals vs. fitted values, normal Q–Q plot, scale–location plot, and residuals vs. leverage) for the fitted linear model. Normality of residuals was additionally evaluated using the Shapiro–Wilk test. The Shapiro–Wilk test did not indicate a significant deviation from normality (W = 0.20, p = .204).


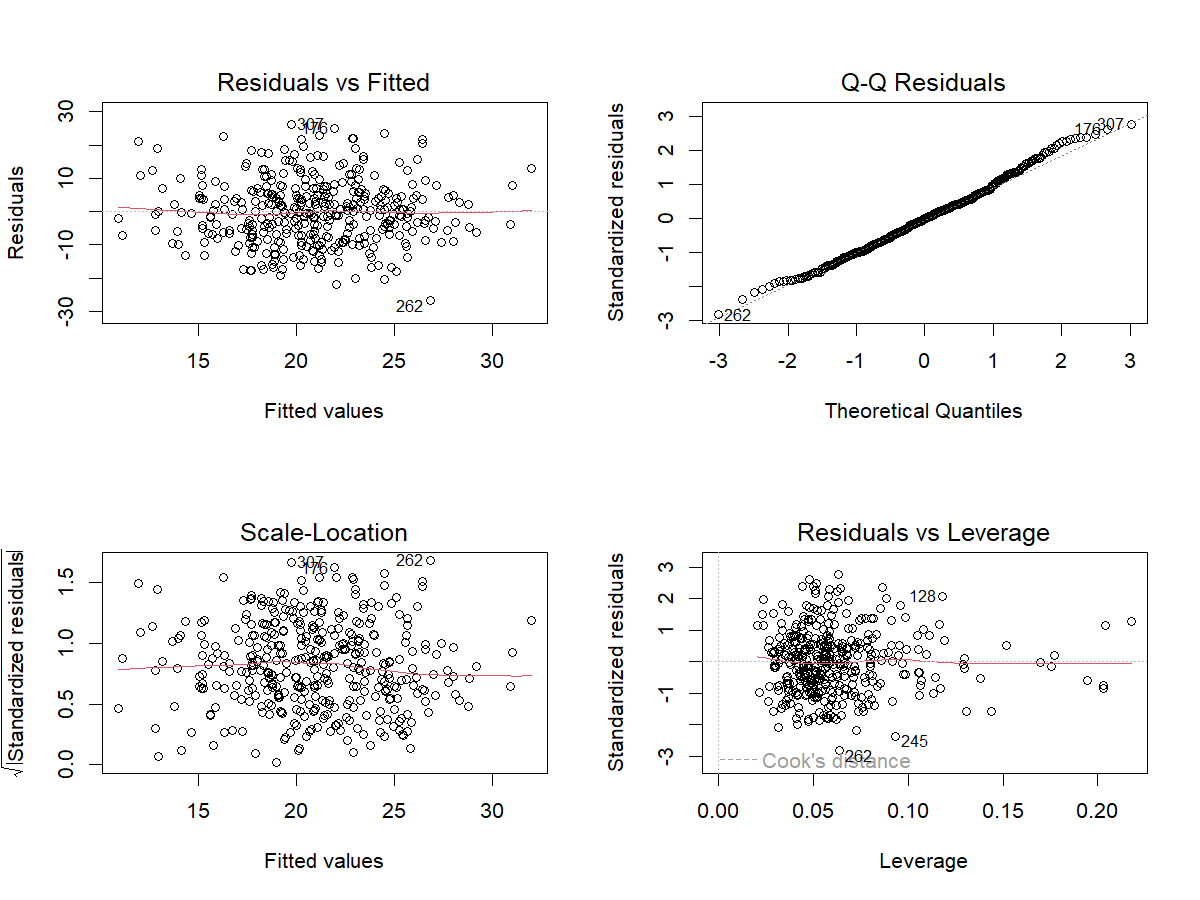


Supplementary Figure 3. Residual diagnostic plots for the Kenny Music Performance Anxiety Inventory-Revised Factor 2

Each panel shows standard residual diagnostic plots (residuals vs. fitted values, normal Q–Q plot, scale–location plot, and residuals vs. leverage) for the fitted linear model. Normality of residuals was additionally evaluated using the Shapiro–Wilk test. The Shapiro–Wilk test did not indicate a significant deviation from normality (W = 0.18, p = .176).


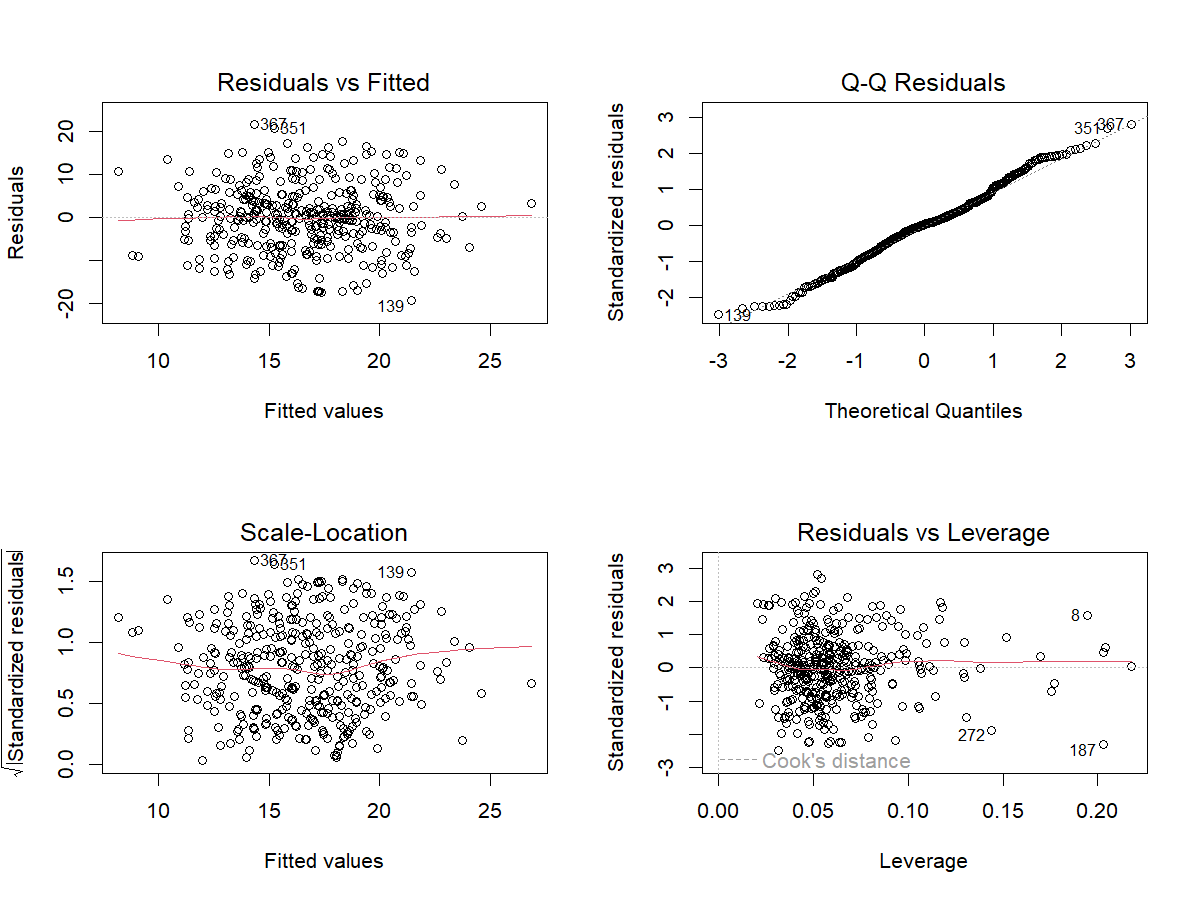


Supplementary Figure 4. Residual diagnostic plots for the Kenny Music Performance Anxiety Inventory-Revised Factor 3

Each panel shows standard residual diagnostic plots (residuals vs. fitted values, normal Q–Q plot, scale–location plot, and residuals vs. leverage) for the fitted linear model. Normality of residuals was additionally evaluated using the Shapiro–Wilk test. The Shapiro–Wilk test did not indicate a significant deviation from normality (W = 0.06, p = .058).

**
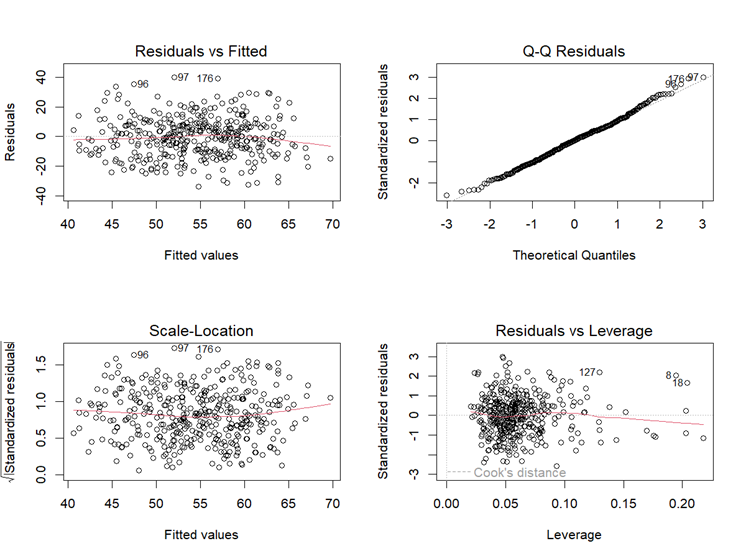
**

Supplementary Figure 5. Residual diagnostic plots for the Performance Anxiety Questionnaire total score

Each panel shows standard residual diagnostic plots (residuals vs. fitted values, normal Q–Q plot, scale–location plot, and residuals vs. leverage) for the fitted linear model. Normality of residuals was additionally evaluated using the Shapiro–Wilk test. The Shapiro–Wilk test did not indicate a significant deviation from normality (W = 0.51, p = .509).

# Supplementary Tables

Supplementary Table 1. Variance inflation factors for predictors

| **Predictor** | **VIF** | **Tolerance** |
| --- | --- | --- |
| Age | 2.91 | 0.34 |
| Start age | 2.81 | 0.36 |
| Years of performance experience | 4.45 | 0.22 |
| Performance count (past 5 years, log) | 1.75 | 0.57 |
| Performance count (lifetime, log) | 2.20 | 0.45 |
| Gender | 1.19 | 0.84 |
| Music-related income | 1.52 | 0.66 |
| Attendance at a music-specialized school | 1.57 | 0.64 |
| Attendance at private lessons | 1.15 | 0.87 |
| Awards | 1.12 | 0.89 |

Note: VIF: variance inflation factor.

Supplementary Table 2. Univariate associations between participant attributes and the Kenny Music Performance Anxiety Inventory-Revised outcomes

| **Attribute** | **Outcome** | **Est.** | **95% CI** | **p** |
| --- | --- | --- | --- | --- |
| Age | Total | -0.56 | [-0.88, -0.24] | **<0.001** |
|  | F1 | -0.27 | [-0.38, -0.16] | **<0.001** |
|  | F2 | -0.15 | [-0.25, -0.06] | **<0.01** |
|  | F3 | -0.09 | [-0.16, -0.01] | **<0.05** |
| Start age | Total | -0.15 | [-0.46, 0.17] | 0.36 |
|  | F1 | -0.08 | [-0.19, 0.03] | 0.16 |
|  | F2 | -0.02 | [-0.12, 0.07] | 0.60 |
|  | F3 | -0.07 | [-0.14, 0.01] | 0.08 |
| Years of performance experience | Total | -0.28 | [-0.52, -0.04] | **<0.05** |
|  | F1 | -0.12 | [-0.20, -0.03] | **<0.01** |
|  | F2 | -0.09 | [-0.16, -0.02] | **<0.01** |
|  | F3 | -0.02 | [-0.07, 0.04] | 0.58 |
| Performance count (past 5 years, log) | Total | -1.42 | [-4.29, 1.44] | 0.33 |
|  | F1 | -0.43 | [-1.44, 0.57] | 0.40 |
|  | F2 | -0.21 | [-1.05, 0.64] | 0.63 |
|  | F3 | 0.27 | [-0.41, 0.95] | 0.44 |
| Performance count (lifetime, log) | Total | -3.16 | [-5.25, -1.07] | **<0.01** |
|  | F1 | -1.22 | [-1.95, -0.48] | **<0.01** |
|  | F2 | -0.87 | [-1.49, -0.26] | **<0.01** |
|  | F3 | -0.16 | [-0.66, 0.34] | 0.53 |
| Gender (Women vs. Men) | Total | 8.59 | [1.33, 15.86] | **<0.05** |
|  | F1 | 3.95 | [1.49, 6.42] | **<0.01** |
|  | F2 | 1.00 | [-1.13, 3.12] | 0.36 |
|  | F3 | 2.24 | [0.52, 3.97] | **<0.05** |
| Music-related income (Yes vs. No) | Total | 8.51 | [1.12, 15.90] | **<0.05** |
|  | F1 | 3.32 | [0.73, 5.90] | **<0.05** |
|  | F2 | 1.90 | [-0.25, 4.06] | 0.08 |
|  | F3 | 2.88 | [1.15, 4.61] | **<0.01** |
| Attendance at a music-specialized school (Yes vs. No) | Total | 15.21 | [8.29, 22.14] | **<0.001** |
|  | F1 | 5.60 | [3.16, 8.04] | **<0.001** |
|  | F2 | 4.10 | [2.07, 6.13] | **<0.001** |
|  | F3 | 3.22 | [1.57, 4.87] | **<0.001** |
| Attendance at private lessons  (Yes vs. No) | Total | 6.19 | [-1.60, 13.98] | 0.12 |
|  | F1 | 2.70 | [0.07, 5.33] | **<0.05** |
|  | F2 | -0.12 | [-2.33, 2.09] | 0.92 |
|  | F3 | 2.81 | [0.99, 4.64] | **<0.01** |
| Awards (Yes vs. No) | Total | 7.71 | [0.64, 14.78] | **<0.05** |
|  | F1 | 4.08 | [1.61, 6.54] | **<0.01** |
|  | F2 | 2.76 | [0.68, 4.84] | **<0.01** |
|  | F3 | 1.23 | [-0.44, 2.89] | 0.15 |
| Primary genre | Total |  | [, ] | 0.10 |
|  | F1 |  | [, ] | **<0.05** |
|  | F2 |  | [, ] | 0.17 |
|  | F3 |  | [, ] | **<0.05** |
| Instrument category | Total |  | [, ] | 0.14 |
|  | F1 |  | [, ] | 0.06 |
|  | F2 |  | [, ] | 0.42 |
|  | F3 |  | [, ] | **<0.05** |
| Performance setting | Total |  | [, ] | 0.10 |
|  | F1 |  | [, ] | **<0.05** |
|  | F2 |  | [, ] | 0.09 |
|  | F3 |  | [, ] | **<0.05** |

Est. denotes the univariate effect estimate. Regarding continuous predictors, the estimate represents the regression slope. Regarding binary predictors, effect estimates correspond to the difference coded according to the label shown in the table header: A vs. B is interpreted as A - B. 95% confidence intervals (CIs) in brackets. Raw p-values are reported. Regarding multi-level predictors, results of an omnibus analysis of variance are shown here.

Supplementary Table 3. Univariate associations between participant attributes and the Performance Anxiety Questionnaire

| **Attribute** | **Est.** | **95% CI** | **p** |
| --- | --- | --- | --- |
| Age | -0.21 | [-0.34, -0.07] | **<0.01** |
| Start age | -0.01 | [-0.14, 0.13] | 0.92 |
| Years of performance experience | -0.13 | [-0.23, -0.03] | **<0.05** |
| Performance count (past 5 years, log) | -1.06 | [-2.26, 0.14] | 0.08 |
| Performance count (lifetime, log) | -1.83 | [-2.70, -0.96] | **<0.001** |
| Gender (Women vs. Men) | 4.14 | [1.16, 7.12] | **<0.01** |
| Music-related income (Yes vs. No) | 3.08 | [0.06, 6.09] | **<0.05** |
| Attendance at a music-specialized school (Yes vs. No) | 7.24 | [4.41, 10.06] | **<0.001** |
| Attendance at private lessons (Yes vs. No) | 4.49 | [1.31, 7.67] | **<0.01** |
| Awards (Yes vs. No) | 2.88 | [-0.12, 5.88] | 0.06 |
| Primary genre |  | [, ] | 0.13 |
| Instrument category |  | [, ] | 0.37 |
| Performance setting |  | [, ] | **<0.05** |

Note: Est. denotes the univariate effect estimate. Regarding continuous predictors, the estimate represents the regression slope. Regarding binary predictors, effect estimates correspond to the difference coded according to the label shown in the table header: A vs. B is interpreted as A - B. 95% confidence intervals (CIs) in brackets. Raw p-values are reported. Regarding multi-level predictors, results of an omnibus analysis of variance are shown here.

Supplementary Table 4. Bootstrap stability results for the score on the Kenny Music Performance Anxiety Inventory-Revised

| **Predictor** | **Inclusion rate (%)** | **Median B** | **95% Bootstrap CI** |
| --- | --- | --- | --- |
| **Attended music-specialized school: Yes (vs. No)** | 98.2 | 12.1 | [4.50, 20.24] |
| **Performance count (all-time, log)** | 83.6 | -3.73 | [-6.17, -1.64] |
| Age | 80 | -0.3 | [-0.64, 0.10] |
| Instrument category: Guitar (vs. Piano) | 55.4 | -7.27 | [-17.68, 4.00] |
| Instrument category: Japanese (vs. Piano) | 55.4 | -22.78 | [-45.19, -0.49] |
| Instrument category: Percussion (vs. Piano) | 55.4 | 10.01 | [-5.66, 23.71] |
| Instrument category: String (vs. Piano) | 55.4 | -7.68 | [-26.12, 9.09] |
| Instrument category: Voice (vs. Piano) | 55.4 | -7.33 | [-16.72, 2.93] |
| Instrument category: Wind (vs. Piano) | 55.4 | -3.77 | [-15.51, 7.26] |
| Performance setting: 10+ (vs. Solo) | 51.6 | 4.79 | [-5.48, 14.79] |
| Performance setting: 2-10 (vs. Solo) | 51.6 | -6.75 | [-13.23, -0.22] |
| Performance setting: Others (vs. Solo) | 51.5 | 16.27 | [-9.01, 38.78] |
| Primary genre: Jazz (vs. Classical) | 43.8 | 1.87 | [-14.73, 20.23] |
| Primary genre: Others (vs. Classical) | 43.8 | -8.44 | [-17.88, 2.22] |
| Primary genre: Pops (vs. Classical) | 43.8 | -0.64 | [-10.26, 10.17] |
| Primary genre: Rock (vs. Classical) | 43.8 | -1.19 | [-12.66, 13.32] |
| Music-related income: Yes (vs. No) | 42.2 | 8.96 | [4.46, 14.82] |
| Years of performance experience | 37.3 | -0.25 | [-0.58, 0.16] |
| Awards: Yes (vs. No) | 36.9 | 6.05 | [2.64, 11.99] |
| Gender: Women (vs. Men) | 33.7 | 5.15 | [0.69, 10.41] |
| Private lessons: Yes (vs. No) | 25.8 | 7.27 | [3.82, 13.55] |
| Performance count (past 5 years, log) | 9.4 | -2.95 | [-5.32, 2.23] |
| Start age | 5.8 | -0.23 | [-0.57, 0.32] |

**Note:** Model stability was evaluated using bootstrap resampling (1,000 iterations). In each resample, the full analytic pipeline was repeated, including univariate screening (p < .05) and forward stepwise selection based on adjusted R². Inclusion rate (%) indicates the proportion of bootstrap samples in which each coefficient was retained in the final model. Median B represents the median unstandardized regression coefficient across resamples in which the term was selected. The 95% bootstrap confidence intervals (Cis) are percentile based and were computed from non-missing bootstrap estimates. Terms that were statistically significant in the original multiple regression model are shown in bold.

Supplementary Table 5. Bootstrap stability results for the score on the Kenny Music Performance Anxiety Inventory-Revised Factor 1

| **Predictor** | **Inclusion rate (%)** | **Median B** | **95% Bootstrap CI** |
| --- | --- | --- | --- |
| **Age** | 98.2 | -0.16 | [-0.29, -0.01] |
| Attended music-specialized school: Yes (vs. No) | 96.9 | 3.34 | [0.83, 6.29] |
| **Performance count (all-time, log)** | 88 | -1.37 | [-2.30, -0.52] |
| Awards: Yes (vs. No) | 74.4 | 2.51 | [1.17, 4.80] |
| Performance setting: 10+ (vs. Solo) | 67.3 | 0.59 | [-2.76, 4.24] |
| Performance setting: 2-10 (vs. Solo) | 67.3 | -2.44 | [-4.87, 0.56] |
| Performance setting: Others (vs. Solo) | 67.2 | 8.33 | [-4.69, 19.29] |
| Instrument category: Guitar (vs. Piano) | 65.4 | -2.84 | [-6.93, 1.06] |
| Instrument category: Japanese (vs. Piano) | 65.4 | -7.69 | [-15.64, 0.30] |
| Instrument category: Percussion (vs. Piano) | 65.4 | 2.38 | [-2.33, 6.94] |
| Instrument category: String (vs. Piano) | 65.4 | -2.93 | [-10.83, 4.19] |
| Instrument category: Voice (vs. Piano) | 65.4 | -1.8 | [-5.26, 1.95] |
| Instrument category: Wind (vs. Piano) | 65.4 | -1.56 | [-5.17, 2.53] |
| Music-related income: Yes (vs. No) | 57.8 | 3.26 | [1.47, 5.72] |
| Primary genre: Jazz (vs. Classical) | 54.9 | -0.72 | [-5.79, 4.76] |
| Primary genre: Others (vs. Classical) | 54.9 | -2.76 | [-5.95, 0.93] |
| Primary genre: Pops (vs. Classical) | 54.9 | 0.21 | [-3.33, 4.14] |
| Primary genre: Rock (vs. Classical) | 54.9 | 0.39 | [-3.81, 4.63] |
| Gender: Women (vs. Men) | 48.6 | 1.55 | [-1.50, 3.89] |
| Private lessons: Yes (vs. No) | 40 | 2.66 | [1.40, 4.79] |
| Years of performance experience | 38.3 | -0.08 | [-0.24, 0.09] |
| Start age | 13.2 | -0.11 | [-0.28, 0.05] |
| Performance count (past 5 years, log) | 9.5 | -1.01 | [-1.99, 1.13] |

**Note:** Model stability was evaluated using bootstrap resampling (1,000 iterations). In each resample, the full analytic pipeline was repeated, including univariate screening (p < .05) and forward stepwise selection based on adjusted R². Inclusion rate (%) indicates the proportion of bootstrap samples in which each coefficient was retained in the final model. Median B represents the median unstandardized regression coefficient across resamples in which the term was selected. The 95% bootstrap confidence intervals (Cis) are percentile based and were computed from non-missing bootstrap estimates. Terms that were statistically significant in the original multiple regression model are shown in bold.

Supplementary Table 6. Bootstrap stability results for the score on the Kenny Music Performance Anxiety Inventory-Revised Factor 2

| **Predictor** | **Inclusion rate (%)** | **Median B** | **95% Bootstrap CI** |
| --- | --- | --- | --- |
| **Attended music-specialized school: Yes (vs. No)** | 97.4 | 3.81 | [1.74, 6.10] |
| **Performance count (all-time, log)** | 76.9 | -0.98 | [-1.68, -0.41] |
| Age | 66.8 | -0.07 | [-0.15, 0.07] |
| Awards: Yes (vs. No) | 56.2 | 1.8 | [0.78, 3.80] |
| Years of performance experience | 56.2 | -0.09 | [-0.19, -0.03] |
| Performance setting: 10+ (vs. Solo) | 51 | 2.25 | [-0.56, 5.05] |
| Performance setting: 2-10 (vs. Solo) | 51 | -2.26 | [-4.35, -0.18] |
| Performance setting: Others (vs. Solo) | 50.9 | -2.66 | [-7.83, 4.87] |
| Primary genre: Jazz (vs. Classical) | 47.5 | 3.43 | [-1.60, 8.06] |
| Primary genre: Others (vs. Classical) | 47.5 | -2.24 | [-4.84, 1.05] |
| Primary genre: Pops (vs. Classical) | 47.5 | 1.16 | [-1.91, 4.05] |
| Primary genre: Rock (vs. Classical) | 47.5 | 0.42 | [-2.89, 3.92] |
| Instrument category: Guitar (vs. Piano) | 35.3 | -1.08 | [-4.55, 2.55] |
| Instrument category: Japanese (vs. Piano) | 35.3 | -6.25 | [-12.73, 0.33] |
| Instrument category: Percussion (vs. Piano) | 35.3 | 3.46 | [-2.41, 8.44] |
| Instrument category: String (vs. Piano) | 35.3 | -0.79 | [-6.16, 3.53] |
| Instrument category: Voice (vs. Piano) | 35.3 | -0.97 | [-3.88, 2.12] |
| Instrument category: Wind (vs. Piano) | 35.3 | -0.07 | [-3.88, 3.28] |
| Music-related income: Yes (vs. No) | 24.8 | 2.27 | [1.19, 4.09] |
| Gender: Women (vs. Men) | 4.8 | 1.43 | [-0.94, 2.71] |
| Performance count (past 5 years, log) | 4.8 | -0.67 | [-1.21, 1.50] |
| Start age | 4.8 | -0.08 | [-0.22, 0.11] |
| Private lessons: Yes (vs. No) | 4.4 | -2.63 | [-3.92, 2.97] |

**Note:** Model stability was evaluated using bootstrap resampling (1,000 iterations). In each resample, the full analytic pipeline was repeated, including univariate screening (p < .05) and forward stepwise selection based on adjusted R². Inclusion rate (%) indicates the proportion of bootstrap samples in which each coefficient was retained in the final model. Median B represents the median unstandardized regression coefficient across resamples in which the term was selected. The 95% bootstrap confidence intervals (CIs) are percentile based and were computed from non-missing bootstrap estimates. Terms that were statistically significant in the original multiple regression model are shown in bold.

Supplementary Table 7. Bootstrap stability results for the score on the Kenny Music Performance Anxiety Inventory-Revised Factor 3

| **Predictor** | **Inclusion rate (%)** | **Median B** | **95% Bootstrap CI** |
| --- | --- | --- | --- |
| Attended music-specialized school: Yes (vs. No) | 81.8 | 1.73 | [0.12, 3.78] |
| Private lessons: Yes (vs. No) | 77.5 | 2.16 | [1.06, 3.86] |
| Instrument category: Guitar (vs. Piano) | 76.3 | -1.53 | [-4.19, 1.02] |
| Instrument category: Japanese (vs. Piano) | 76.3 | -5.52 | [-11.00, -0.46] |
| Instrument category: Percussion (vs. Piano) | 76.3 | 2.54 | [-1.15, 6.20] |
| Instrument category: String (vs. Piano) | 76.3 | -2.12 | [-6.29, 1.63] |
| Instrument category: Voice (vs. Piano) | 76.3 | -0.96 | [-3.19, 1.31] |
| Instrument category: Wind (vs. Piano) | 76.3 | -0.22 | [-3.13, 2.61] |
| Music-related income: Yes (vs. No) | 74.2 | 2.19 | [1.00, 4.00] |
| Performance setting: 10+ (vs. Solo) | 72.8 | 0.99 | [-1.82, 3.66] |
| Performance setting: 2-10 (vs. Solo) | 72.8 | -1.59 | [-3.33, 0.26] |
| Performance setting: Others (vs. Solo) | 72.7 | 8.05 | [0.30, 13.69] |
| Age | 53.8 | -0.07 | [-0.14, -0.02] |
| Primary genre: Jazz (vs. Classical) | 42.6 | -0.82 | [-4.46, 3.21] |
| Primary genre: Others (vs. Classical) | 42.6 | -1.34 | [-3.54, 1.96] |
| Primary genre: Pops (vs. Classical) | 42.6 | -0.72 | [-2.92, 2.22] |
| Primary genre: Rock (vs. Classical) | 42.6 | -0.64 | [-3.71, 2.54] |
| Gender: Women (vs. Men) | 38.8 | 0.9 | [-1.29, 2.21] |
| Awards: Yes (vs. No) | 24.2 | 1.56 | [0.88, 2.96] |
| Start age | 12 | -0.04 | [-0.09, 0.06] |
| Performance count (all-time, log) | 7.9 | -0.83 | [-1.19, -0.25] |
| Performance count (past 5 years, log) | 7.1 | 0.56 | [-0.98, 1.17] |
| Years of performance experience | 5 | -0.07 | [-0.14, -0.01] |

**Note:** Model stability was evaluated using bootstrap resampling (1,000 iterations). In each resample, the full analytic pipeline was repeated, including univariate screening (p < .05) and forward stepwise selection based on adjusted R². Inclusion rate (%) indicates the proportion of bootstrap samples in which each coefficient was retained in the final model. Median B represents the median unstandardized regression coefficient across resamples in which the term was selected. The 95% bootstrap confidence intervals (CIs) are percentile based and were computed from non-missing bootstrap estimates. Terms that were statistically significant in the original multiple regression model are shown in bold.

Supplementary Table 8. Bootstrap stability results for the score on the Performance Anxiety Questionnaire

| **Predictor** | **Inclusion rate (%)** | **Median B** | **95% Bootstrap CI** |
| --- | --- | --- | --- |
| **Attended music-specialized school: Yes (vs. No)** | 99.9 | 6.03 | [3.22, 9.25] |
| **Performance count (all-time, log)** | 98.6 | -1.93 | [-3.04, -0.88] |
| Private lessons: Yes (vs. No) | 70.4 | 3.65 | [1.76, 6.77] |
| Age | 61.3 | -0.12 | [-0.26, 0.10] |
| Gender: Women (vs. Men) | 56.1 | 2.43 | [0.90, 4.83] |
| Performance setting: 10+ (vs. Solo) | 52.5 | -2.36 | [-5.63, 2.43] |
| Performance setting: 2-10 (vs. Solo) | 52.5 | -2.55 | [-5.47, 1.26] |
| Performance setting: Others (vs. Solo) | 52.4 | 7.45 | [-6.88, 21.48] |
| Primary genre: Jazz (vs. Classical) | 44.3 | -1.87 | [-6.63, 3.31] |
| Primary genre: Others (vs. Classical) | 44.3 | -2.47 | [-6.02, 1.98] |
| Primary genre: Pops (vs. Classical) | 44.3 | 1.71 | [-3.27, 6.13] |
| Primary genre: Rock (vs. Classical) | 44.3 | 2.42 | [-3.11, 7.76] |
| Instrument category: Guitar (vs. Piano) | 42.6 | -1.4 | [-5.83, 4.35] |
| Instrument category: Japanese (vs. Piano) | 42.6 | -6.51 | [-15.18, 2.83] |
| Instrument category: Percussion (vs. Piano) | 42.6 | 2.28 | [-4.05, 8.50] |
| Instrument category: String (vs. Piano) | 42.6 | -7.54 | [-18.68, 2.15] |
| Instrument category: Voice (vs. Piano) | 42.6 | -2.48 | [-6.49, 1.56] |
| Instrument category: Wind (vs. Piano) | 42.6 | -1.68 | [-6.20, 3.30] |
| Years of performance experience | 33.3 | -0.1 | [-0.23, 0.07] |
| Music-related income: Yes (vs. No) | 32.6 | 3.03 | [1.63, 5.16] |
| Awards: Yes (vs. No) | 30.1 | 2.54 | [1.28, 4.97] |
| Performance count (past 5 years, log) | 14 | -1.03 | [-1.92, 1.07] |
| Start age | 2 | -0.1 | [-0.18, 0.23] |

**Note:** Model stability was evaluated using bootstrap resampling (1,000 iterations). In each resample, the full analytic pipeline was repeated, including univariate screening (p < .05) and forward stepwise selection based on adjusted R². Inclusion rate (%) indicates the proportion of bootstrap samples in which each coefficient was retained in the final model. Median B represents the median unstandardized regression coefficient across resamples in which the term was selected. The 95% bootstrap confidence intervals (CIs) are percentile based and were computed from non-missing bootstrap estimates. Terms that were statistically significant in the original multiple regression model are shown in bold.
